# An in-home rehabilitation program for the treatment of urinary incontinence symptoms in endometrial cancer survivors: a single-case experimental design study

**DOI:** 10.1007/s00192-021-04981-x

**Published:** 2021-09-25

**Authors:** Stéphanie Bernard, Linda McLean, Samantha Boucher, Luc J. Hébert, Marie Plante, Jean Grégoire, Alexandra Sebastianelli, Marie-Claude Renaud, Marie-Anne Froment, Hélène Moffet

**Affiliations:** 1 Rehabilitation Department, Faculty of Medicine, Université Laval, Québec, Québec, Canada; 2 School of Rehabilitation Sciences, Faculty of Health Sciences, University of Ottawa, Ottawa, Canada; 3 Hôtel-Dieu de Québec, Department of Gynecologic Oncology, Centre Hospitalier Universitaire de Québec, Québec, Québec Canada; 4 Hôtel-Dieu de Québec, Department of Radiation Oncology, Centre Hospitalier Universitaire de Québec, Québec, Québec Canada

**Keywords:** Gynecological cancer, Cancer survivorship, Urinary incontinence, Pelvic floor muscle training, Mobile technology, Bladder training, Physiotherapy

## Abstract

**Introduction and hypothesis:**

There is a high prevalence of urinary incontinence among endometrial cancer survivors. They are also known to present with pelvic floor muscle alterations. Evidence on the effects of conservative interventions for the management of UI is scarce. This study aims at verifying the effects of an in-home rehabilitation program, including the use of a mobile technology, to reduce UI severity in endometrial cancer survivors.

**Methods:**

This study used a single-case experimental design with replications. Primary outcome for UI severity was the pad test, and secondary outcomes were the ICIQ-UI SF questionnaire and 3-day bladder diary. Pelvic floor muscle function was assessed using 2D-transperineal ultrasound and intravaginal dynamometry. Adherence was documented using mobile technology and an exercise log. Visual and non-parametric analyses of longitudinal data were conducted.

**Results:**

Results show a reduction in UI severity for 87.5% of participants, with a significant relative treatment effect of moderate size (RTE: 0.30). Significant small relative treatment effects were found for the quick contraction and endurance dynamometric tests.

**Conclusion:**

This study provides new evidence that endometrial cancer survivors can improve the severity of their UI following an in-home rehabilitation program, including the use of a mobile technology. This mode of delivery has the potential to address a gap in access to pelvic floor physiotherapy services for survivors of EC living in rural and remote communities.

## Introduction

Endometrial cancer (EC) is the fifth most prevalent cancer in women worldwide [1]. The number of survivors living with the physical consequences of this cancer and its treatments is higher than ever [2]. Urinary incontinence (UI) and urgency have been frequently reported after the treatment of EC by surgery and radiation therapy. Prevalence rates are as high as 40% to 83% for this population [3]. With symptoms of UI reported in circumstances of both physical exertion and urgency, UI is known to significantly reduce health-related quality of life in survivors of gynecological cancers [4].

Impaired strength, endurance and ability to contract rapidly have been reported in the pelvic floor muscles (PFMs) among women with UI who have no history of gynecological cancer [5]. A recent Cochrane systematic review of 31 trials demonstrated that pelvic floor muscle training (PFMT) can improve or cure urinary symptoms, supporting a longstanding recommendation for PFMT as a first-line treatment for UI [6]. Lifestyle modifications and bladder training are also recommended as conservative interventions for UI and urgency in women without cancer [7]. These interventions may include education on fluid consumption, diet and scheduled voiding regimens.

PFM impairments have also been described in survivors of gynecological cancers with UI, namely lower strength and power of the PFMs, with worst urinary symptoms seen in women with poorer endurance [8]. Rehabilitation programs including PFMT for women with UI after EC have been poorly studied. A recent review published by Brennen and colleagues (2020) synthesized the effects of PFM therapies for pelvic floor dysfunctions after gynecological cancer treatments [9]. In two of the retrieved articles that have studied the effects of PFMT on UI, patient-reported outcomes were not significantly improved in either [10, 11]. In one of these studies, led by Yang et al. (2012), it remains unclear whether the intervention group had any symptoms of UI before the intervention, which could explain why symptoms of UI were not improved [11]. In the other study, conducted by Rutledge and colleagues (2014), an in-home PFMT program was not found to improve the impact of UI on quality of life. However, significant changes were reported for patients' perceived improvement of their condition and in the number of women with moderate to severe UI [10]. The state of current knowledge remains considerably insufficient to confirm whether a rehabilitation program comprising PFMT and bladder training could reduce UI and urgency in EC survivors [10, 11].

However, access to pelvic health physiotherapy services remains limited, especially for women living in remote areas. Given the high proportion of women with UI following gynecological cancer [12], it is relevant to consider a rehabilitation approach that could address these gaps in services. Using mobile technology to assist with the conservative management of UI has been proposed as a modern approach to address these issues [13]. As such, the aim of this study was to measure the short-term effects of a 12-week in-home rehabilitation program, including the use of mobile technology (the Elvie Trainer), on the severity of UI among survivors of EC. We hypothesized that more than half of the women who completed the program would present with a reduction in the severity of UI after the program, as measured by the primary outcome measure, a standardized pad test.

## Materials and methods

### Design

**Fig. 1:**
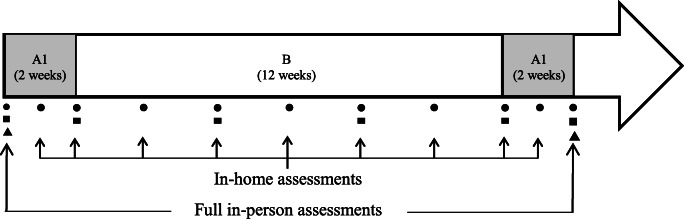
Study design and flow of assessments. Types of outcomes collected at each assessment are illustrated with symbols: ● measurement of the primary urinary outcome (pad test); ■ measurement of secondary urinary outcomes (ICIQ-UI SF and 3-day bladder diary); ▲ measurement of the PFM outcomes (dynamometry and ultrasound imaging)

A single-case experimental design (three phases; A1-B-A2) with replications was used to explore the effects of the rehabilitation program at the individual level. Although it is recommended to replicate the intervention with three to five participants in this type of study [14], we opted for a sample of eight participants because of the high incidence of this population. Repeated assessments (*n* = 11) were conducted over a 16-week period: (A1) a pre-intervention phase (2 weeks) where baseline assessments were conducted, (B) an intervention phase (12 weeks) including an in-home rehabilitation program with weekly telephone follow-up by a physiotherapist and (A2) a post-intervention phase (2 weeks) where the short-term effects were evaluated. A flow chart of the protocol is illustrated in Fig. 1. Approval for this study was obtained from the Ethics Committee of the CHU-Université Laval.

### Participants

Eligible women had been treated with total hysterectomy and vault brachytherapy for endometrial cancer stage I at least 12 months prior to participating, were continent prior to the cancer treatments and presented with de novo UI at least three times per week based on self-report. Exclusion criteria for participation were having pelvic organ prolapse ≥ POP-Q stage 2, urinary or vaginal infection at medical follow-up, severe vaginal stenosis (unable to receive a bi-digital assessment), concomitant cancer, other adjuvant cancer treatment (including external-beam radiation therapy) or any cognitive or physical impairments that would limit participation in this study. Women were invited to participate by their radio-oncologist, posters at Hotel-Dieu de Québec and invitation letters sent out by the medical records department. All participants provided written informed consent.

### Intervention

The rehabilitation program included three main components: a PFMT program, bladder training regimen and counseling on lifestyle habits. The PFMT was accomplished in conjunction with the Elvie Trainer and its mobile application (mApp). The Elvie Trainer is an egg-shaped intra-vaginal dynamometer that connects wirelessly to its mApp to provide real-time biofeedback. An Elvie Trainer was given to each participant, and an iPad mini was provided for the duration of the program. Participants received their daily exercise program through the mApp. Weekly telephone follow-ups were carried out by a physiotherapist to offer personalized advice on exercise variety and positions for training [7]. For the last two components, motivational counseling was given during the weekly follow-ups to teach urge suppression techniques, bladder emptying techniques, mindfulness exercises and lifestyle choices that may influence bladder symptoms. Short educational videoclips were also available on the iPad for consultation when participants had a question.

### Outcomes

In addition to personal characteristics of the participants, three categories of outcomes were measured: UI outcomes, PFM outcomes (including dynamometric and ultrasound variables) and adherence. The primary UI outcome (pad test) was assessed 11 times, while secondary UI outcomes were recorded 6 times. The dynamometric and ultrasound variables were conducted in person at the Hotel-Dieu de Québec site of the CHU-Université Laval (see Fig. 1). The UI assessments were conducted over the phone by an independent evaluator, using detailed and standardized instructions, at a similar day and time. The evaluator was unaware of when the intervention began or ended and the participant’s evolution.

UI outcomes: the primary outcome was the 1-h pad test, which was conducted by first putting on a pad that had been pre-weighed on a high-precision scale (0.01 g). Then, participants drank a standardized volume of water (750 ml) within 15 min. One hour later, the test was started if there was a desire to void; otherwise, an additional 15- to 30-min wait was granted. Participants performed standardized tasks following instructions given by the evaluator: standing up from a chair (15 times), coughing forcefully (10 times), stepping on the spot vigorously (1 min), jumping jacks (15 times), bending to pick up a pen from the floor (10 times) and washing their hands under running water (1 min) [15]. The pad was weighed after the test, and the difference was calculated to quantify urinary leakage. A practice test was conducted during the hospital-based assessment, but all recorded tests were carried out at home. Secondary UI outcomes included the International Consultation Incontinence Questionnaire for Urinary Incontinence-Short-Form(ICIQ-UI SF) and a 3-day bladder diary, both recognized as valid, reliable and responsive tools to assess UI [16–19].

PFM outcomes: after voiding the bladder, participants were positioned in the lithotomy position and instructed about performing a correct PFM contraction, which was confirmed through bidigital palpation. Then, an automated portable lightweight intra-vaginal dynamometer was inserted and used to assess PFM function [20]. The two 3D-printed arms, covered with a medical glove and lubricated, were aligned comfortably along the participant’s vaginal canal. Four tests were conducted: (1) passive test: five trials of opening the dynamometer arms to 10 mm at 10 mm/s speed while the PFMs were kept at rest; (2) maximal voluntary contraction (MVC): three trials of voluntarily squeezing the PFMs as strongly as possible for 10 s; (3) quick contractions: three trials of repeatedly contracting and relaxing the PFM as strongly and quickly as possible during a 10-s interval; (4) endurance: one trial of holding a maximal contraction as long as possible or until 50% of the MVC has been reached. Ultrasound imaging was then used to assess PFM morphology. Using an Aplio 500 with a two-dimensional 3–7-Mhz curved-array transducer, measures of the levator plate length were made in the mid-sagittal plane with the PFMs at rest, during an MVC and during a maximal Valsalva maneuver, and urethral length was measured at rest, as described elsewhere [21].

To track adherence, the Elvie mApp records each session performed with the Trainer, documenting progression and training sessions, and these data were extracted at the end of the study. In addition, the participants were invited to write down the exercises performed in an exercise log, which was also collected after the study. Technical issues encountered were also documented. Adherence to the program was promoted by the therapist using various adherence promotion strategies [22], which were documented at every telephone session. Lastly, using a semi-structured interview format, two open-ended questions were asked after the study to examine participant’s satisfaction with the care received and confidence in pursuing the program on her own afterwards.

### Analysis

Descriptive analyses were conducted on the whole sample for outcomes at baseline expressed as mean and standard deviations. Following the method of Lane and Gast [23], visual analyses of graphic displays were used to compare pad test results from the A2 and B phases with the baseline phase. Changes in absolute levels were investigated, which involved calculating the difference between the final and initial data in each phase and then comparing between phases. Changes in relative levels were also calculated, in which the median of the first half of a phase was subtracted from the median of the second half of that phase. A difference > 1.4 g of urine leakage on the pad test was needed to be considered as a change between phases [15]. Trend analysis was also conducted; curves were calculated for each phase, and directional changes were used to compare between phases. Finally, stability of the data was demonstrated when 80% of data points were included in a stability envelope representing ± 25% of the median value of each phase [23]. Nonparametric analyses of longitudinal data in factorial experiments (nparLD) were conducted on all UI and PFM outcomes for comparison between the A2 and A1 phases for the whole group. All trials were included. Threshold values of *p* < 0.05 and a relative treatment effect (RTE) ≠ 0.50 were used to identify significance, meaning that the null hypothesis could not be rejected with a RTE = 0.50. For interpretation of the RTE values, benchmarks from Vargha and Delaney were followed [24]: small increasing effects with RTE values between 0.56 and 0.63, moderate effects between 0.64 and 0.70 and large effects for values ≥ 0.71; for decreasing effects, small effects with RTE values between 0.44 and 0.37, moderate effects between 0.36 and 0.30 and large effects for values ≤ 0.29. Analyses were conducted using Microsoft Excel (v16.42, Microsoft 2020), SPSS Statistics version 25 (IBM Corp, Armonk, NY) and R Studio (v4.0.2, R Foundation for Statistical Computing, 2020).

## Results

Invitation letters (*n* = 416) were sent over the course of a 12-month period (July 2018 to June 2019). *N* = 38 potential participants contacted the research team, and *n* = 8 were found eligible and agreed to participate. Reasons for non-eligibility were: having urinary incontinence prior to the cancer (*n* = 11), not having at least three episodes of leakage per week (*n* = 9), having other medical treatments preventing participation (*n* = 6) and not interested in participating (*n* = 4). There were no dropouts, and all participants completed all planned assessments, except assessment 8 for participant 8 (S8) because of a temporary illness. Personal characteristics of participants are presented in Table 1. All women reported symptoms of mixed UI, confirmed by the baseline ICIQ-UI SF and 3-day bladder diary, with 1 to 30 episodes of urgency identified. According to the baseline pad tests, UI severity was mild for *n* = 5 (< 10 g) and moderate in *n* = 3 (between 10 g and 20 g) [25].

**Table 1:** Personal characteristics of each participant at baseline

Participants	Age	Body mass index	Parity	Time since EC^a^ (months)	Physical activity per week^b^	Weekly use of dilator (yes / no)	Years with smart phone/tablet^c^	Comfort with technology^d^
S1	39	28.2	0	55	> 5	No	8	Excellent
S2	69	27.9	1	55	1–2	No	8	Moderate
S3	66	23.0	0	55	3–5	No	5	Excellent
S4	76	28.5	1	19	1–2	No	1	Poor
S5	75	26.6	2	24	0	No	0	Poor
S6	70	50.3	1	36	1–2	No	8	High
S7	76	35.8	2	55	1–2	No	4	Moderate
S8	58	24.7	0	44	0	No	3	Moderate

*EC* endometrial cancer, *S1 to S8* participants 1 to 8

^a^Time passed since the end of cancer treatments

^b^Number of times per week that moderate intensity exercise is practiced for at least 20 min: < 1, 1–2, 3–5 and > 5 times a week

^c^Number of years owning a smart phone or tablet

^d^Self-perceived level of comfort using smart phone/tablet technologies among poor, moderate, high and excellent

### Urinary continence outcomes

**Fig. 2:**
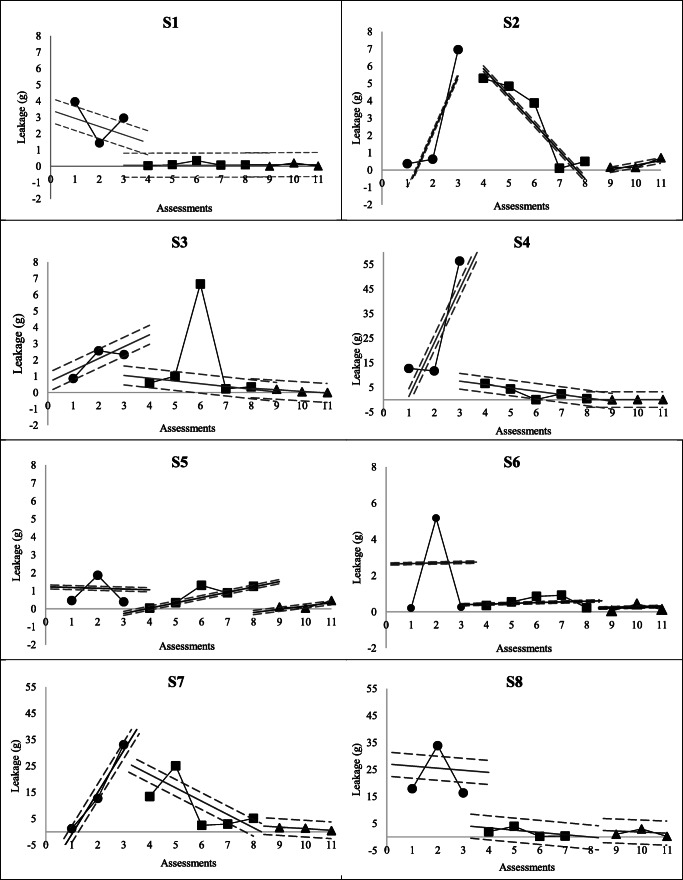
Pad test results with trend lines and stability envelopes for each participant. Profiles of the pad test results with trend lines and stability envelopes (in grams, lower values indicate improvement) of the eight participants (S1 to S8) over the three phases of the study: ● results from A1 phase (baseline); ■ results from B phase (intervention); ▲ results from A2 phase (post-intervention). A descending slope indicates a decrease in pad test results over the corresponding phase, which can indicate improvement. A stability envelope encompassing 80% of data is considered stable. Participants S4, S7 and S8 had more severe urinary incontinence at baseline, explaining the different scale of the y-axes for these participants to increase visual appreciation

Pad test results for each participant are illustrated in Fig. 2 and visual analysis calculations in Table 2. An improvement in absolute and relative levels of leakage was found in six participants (S1–S4, S7–S8) between the A2 and A1 phases. Four of these six participants (S2–S4, S7) also had improved direction of the trendlines, as seen in Fig. 2. Five participants (S1, S3–S4, S7–S8) had improved stability of data during the A2 phase compared with the A1 phase. For stability of trendlines, improvements were identified in all but two participants (S3 and S6). For participant S6, improvements were only observed for the relative level of leakage, while it was only for stability of the trendlines for participant S5. Overall, all but one participant showed improvements regarding level of data (S5).

**Table 2:** Between-phase analysis of absolute change, relative change and stability of the results of the pad tests of each participant (*n* = 8)

Participants	Change from A1 to B		Change from A1 to A2		% of pad tests in the stability envelope^c^		
	Absolute change level (g)^a^	Relative change level (g)^b^	Absolute change level (g)^a^	Relative change level (g)^b^	A1	B	A2
S1	**−2.92**	**−2.12**	**−2.96**	**−2.09**	33.3	**100**	**100**
S2	**−1.66**	1.28	**−6.80**	**−3.65**	33.3	20	66.7
S3	**−1.74**	**−1.65**	**−2.13**	**−2.33**	66.7	**80**	**100**
S4	**−49.80**	**−28.57**	**−56.39**	**−34.00**	66.7	**80**	**100**
S5	−0.35	−0.93	−0.28	−1.06	66.7	20	66.7
S6	0.09	**−2.27**	−0.20	**−2.47**	66.7	20	66.7
S7	**−19.72**	**−3.68**	**−31.52**	**−21.43**	33.3	60	**100**
S8	**−14.33**	**−22.12**	**−15.32**	**−23.09**	66.7	**100**	**100**

*S1 to S8* participants 1 to 8, *A1* baseline phase, *B* intervention phase, *A2*post-intervention phase

^a^Value of the last result minus the first result of the phase. A negative value means that the pad test was lower at the end of the phase, which can indicate improvement

^b^Median value of the second half of the phase minus the median of the first half of the phase. A negative value means that the pad test results were lower in the second half of the phase, which can indicate improvement

^c^Percentage of the total number of pad test results conducted in the phase that was included in the stability envelope (median value of all pad tests ± 25%). Stability criterion is met if ≥ 80% of data are within the stability envelope

Significant improvements are identified in bold

**Fig. 3:**
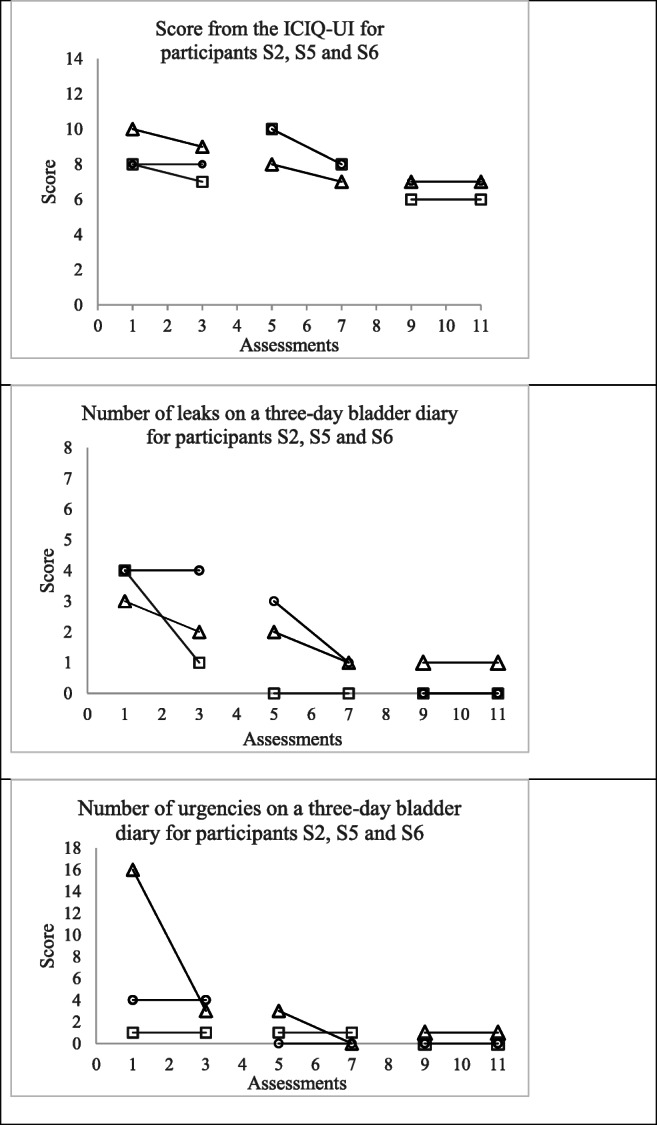
Results from the International Consultation on Incontinence Questionnaire and the 3-day bladder diary for participants S2, S5 and S6. Profiles of the results from the International Consultation on Incontinence Questionnaire (score between 0 and 21; lower values indicate better urinary function) and the 3-day bladder diary (number of events; lower values indicate better urinary function) for participants S2 (□), S5 (○) and S6 (∆) over the three phases of the study (2 assessments in each phase)

Furthermore, as it was not possible to produce three positive pad tests for participants S2, S5 and S6 during the baseline phase, the results from the secondary urinary outcomes were plotted in Fig. 3 to have a deeper look into changes captured by these measures. The ICIQ-UI scores appear highly variable throughout the three phases of the study and do not indicate improvements for either participant. The number of leaks on the 3-day bladder test improved from three (S6) and four leaks (S2–S5) to one leak (S6) and no leak at all (S2–S5) for an equivalent period of time. The number of urgencies recorded on the diary also suggests improvements for participants S5 and S6, although more variability was found for the latter.

Results from the group nonparametric analyses related to UI outcomes are reported in Table 3. All UI outcomes presented significant changes between the A2 and A1 phases, presenting moderate relative treatment effects (RTE) for the reduction of urinary leakage on the pad test (RTE: 0.30) of the ICIQ-UI SF scores (RTE: 0.31) and the number of leaks (RTE: 0.31) and urgencies (RTE: 0.33) on a 3-day bladder diary. The RTE for the number of voids on the 3-day bladder diary was significant but small (RTE: 0.39).

**Table 3:** Results of nonparametric analyses of longitudinal data in factorial experiments (nparLD) for all urinary continence, pelvic floor muscle function and pelvic floor morphology variables between the A2 and A1 phases for the whole sample

	A1^a^	A2^a^	p^b^	RTE^c^	
				A1	A2
Urinary continence outcomes					
Pad test (g)	9.4	0.4	**< 0.0001**	**0.70**	**0.30**
Bladder diary: *n* of leaks	3.8	0.6	**< 0.0001**	**0.69**	**0.31**
Bladder diary: *n* of urges	7.4	1.1	**< 0.0001**	**0.67**	**0.33**
Bladder diary: *n* of voids	23.5	19.7	**0.04**	**0.61**	**0.39**
ICIQ-UI SF (score)	9.3	6.3	**0.0003**	**0.69**	**0.31**
PFM outcomes: **ultrasound variables**					
LP-R (mm)	55.9	53.6	0.11	0.55	0.45
LP-MVC (mm)	44.7	43.9	0.73	0.52	0.48
LP-MVM (mm)	61.7	59.2	0.22	0.54	0.46
UL (mm)	26.0	28.1	0.18	**0.41**	**0.59**
PFM outcomes: **dynamometric variables**					
Passive resistance test					
Baseline (range, *N*)	1.08	1.23	0.45	0.47	0.53
Maximal relative peak (*N*)	4.87	4.71	0.90	0.49	0.51
Decline between maximal and final relative peaks (%)	−23.99	−22.42	0.42	0.46	0.54
Stiffness (*N*/mm)	0.49	0.47	0.90	0.49	0.51
Maximal voluntary contraction test					
Maximal relative force (*N*)	5.75	6.91	0.09	0.45	0.54
Rate of force development (*N*/s)	11.28	14.52	**0.05**	**0.44**	**0.56**
**Quick contraction test**					
No. of peaks	5.6	5.3	0.53	0.48	0.52
Maximal relative force (*N*): First contraction Mean of all included contractions					
	4.44 4.05	6.65 5.51	**0.0001** **0.0007**	**0.42** **0.41**	**0.57** **0.58**
Rate of force development (*N*/s): First contraction Mean of all included contractions					
	12.66 11.88	19.27 17.73	**0.0006** **< 0.0001**	**0.40** **0.40**	**0.60** **0.60**
Endurance test					
Relative peak (*N*)	5.14	7.94	**< 0.0001**	**0.40**	**0.60**
Time for the duration > 50% MVC (s)	5.70	10.53	0.41	0.46	0.54
Area under the curve > 50% MVC (N*s)	14.15	35.49	**0.05**	**0.44**	**0.56**

*A1* baseline phase, *A2*post-intervention phase, *RTE* relative treatment effect, *ICIQ-UI SF* International Consultation Incontinence Questionnaire for Urinary Incontinence-short form, *LP-R* levator plate length at rest, *LP-MVC* levator plate length during a maximal voluntary contraction, *LP-MVM* levator plate length during a maximal Valsalva maneuver, *MVC* maximal voluntary contraction

^a^Expressed as the mean of all included trials

^b^Significant change is identified in bold and corresponds to *p* values < 0.05 derived from nonparametric analyses of longitudinal data in factorial experiments for A1 and A2 periods, including all trials

^c^Significant effect of intervention is identified in bold and corresponds to RTE values derived from nonparametric analyses of longitudinal data in factorial experiments: small increasing effects with RTE values between 0.56 and 0.63, moderate effects between 0.64 and 0.70 and large effects for values ≥ 0.71. For decreasing effects, small effects are identified with RTE values between 0.44 and 0.37, moderate effects between 0.36 and 0.30 and large effects for values ≤ 0.29

### PFM outcomes

Results from the nonparametric group analyses related to the PFM outcomes are also presented in Table 3. No significant RTE was found for the ultrasound variables collected while significant effects were identified for several dynamometric variables. For the MVC test, a significant small RTE was found for the rate of force development (mean A1: 11.28 N/s; mean A2: 14.52 N/s), but not for the peak force itself. For the quick contraction test, a significant small RTE indicating an increase in the relative peak force was found for the first contraction (mean A1: 4.44 N; mean A2: 6.65 N) and for the mean of all contractions (mean A1: 4.05 N; mean A2: 5.51 N). Similarly, a significant small RTE was found for the rate of force development for the first contraction (mean A1: 12.66 N/s; mean A2: 19.27 N/s) and for the mean of all contractions (mean A1: 11.88 N/s; mean A2: 17.73 N/s). The number of contractions during the 10-s interval was not different between phases. For the endurance test, a significant small RTE was found for the area under the curve (mean A1: 14.15 N*s; mean A2: 35.49 N*s), but not for the duration of the contraction.

### Adherence and satisfaction

The total number of training sessions recorded by Elvie mApp ranged between 24 to 124 sessions per participant (mean number of sessions per week: 5.6), with five participants concurrently reporting these sessions on their exercise log. S1 was the participant with the lowest number of weekly sessions on average (*n* = 2), while S2 had the highest (*n* = 10.3). Throughout all telephone follow-ups, the three most frequent adherence promotion strategies used by the therapist were (1) providing information on benefits and consequences, (2) providing general encouragement and (3) providing feedback on performance [22]. Regarding satisfaction, all participants (8/8) responded that they were “satisfied” with the care received, and all (8/8) believed they “had everything in hand” to pursue their exercises on their own once the program was completed.

### Technical issues

Three participants had technical issues while using the mobile technology, all of which were difficulties in connecting the Elvie device to the mApp through a Bluetooth connection. Due to these technical issues, these participants missed part of their training using the Elvie mApp, meaning they practiced the exercises with the device inserted, but without using the biofeedback (S1, 4 weeks; S4, 5 weeks; S7, 2 weeks).

## Discussion

To our knowledge, this is the first study to use a single-case experimental design to measure the effects of a novel in-home rehabilitation program, including the use of mobile technology and remote supervision, on UI severity among survivors of EC. The results indicate that UI severity improved in seven out of eight participants, easily meeting our hypothesis that at least half of the sample would show improvements. Individual improvements were identified through visual analysis calculations. Group analysis confirmed these findings and further identified significant changes in secondary UI outcomes in ICIQ-UI SF scores and in the number of leaks, urgency episodes and voids in the 3-day bladder diary.

Although the intervention and outcomes differed, our results appear to be in line with some of the findings presented by Rutledge and colleagues [10]. In their study, 80% of participants (*n* = 20) reported that their UI was “much better” or “very much better” after a home program of PFMT, and their Incontinence Severity Index score was improved by 5 points [10]. Their program shared similarities with ours regarding the intensity and duration of the PFMT and the education on lifestyle and fluid management [7]. The approach used in the present study differed by including weekly telephone supervision by a physiotherapist. The additional and individualized education provided through these follow-ups, the bladder training and adherence promotion strategies may have driven the favorable outcomes for UI severity in our participants.

With access to pelvic floor physiotherapists being limited, especially for women living in remote areas, it has been suggested that mobile technologies may prove helpful in delivering conservative management for UI [13]. This study proposes a rehabilitation program that includes the use of a mobile technology: the Elvie Trainer device. This technology presents various features that are recommended to improve rehabilitation outcomes: educational features, reinforcements, reminders and self-monitoring of progress [13]. Nevertheless, three of our participants experienced technical issues. The support of the treating physiotherapist was deemed essential in proposing solutions to overcome these difficulties and in giving instructions to continue the exercise program. As such, these results support an in-home rehabilitation approach using a mobile technology, but they also emphasize the importance of including remote supervision by a healthcare professional. The facts that there was no dropout from the program and that overall satisfaction was achieved further support this combined approach as a mean to help numerous women reporting UI after EC treatments.

The PFMT program proposed in this study followed recommended training principles in terms of frequency, intensity, variety of exercises and loading principles [7, 26]. The program was adhered to by most participants, with all but one meeting expectations and practicing 5 days a week during a 12-week period. Previous knowledge indicates that changes in PFM function or morphometry can be expected in women with stress UI after 12 weeks of PFMT [27]. Our results indicate that participants in this study showed improvement in several parameters of PFM function, especially in the quick contraction and endurance tests. With previous findings indicating worst UI symptoms in EC survivors with slower contractions and lower endurance, the importance of not overlooking these aspects during training is highlighted by these results [8].

Despite this, changes in PFM function measured by dynamometry presented small RTEs, and no significant changes in PFM morphology were found by ultrasound imaging. A few reasons could explain why only small effects were identified. First, our sample comprised women who had undergone vaginal brachytherapy and as such damage to the connective tissues, muscles or nerves may have decreased the capacity for the PFMs to improve contractility, as measured by dynamometry, or to lengthen or shorten, as measured by ultrasound imaging [8, 28]. Indeed, radiotherapy is known to cause infiltration of muscle tissue with collagen, resulting in progressive fibrosis that can impact nerve conduction [29]. Hence, it is possible that fibrosis and alterations in neural conduction could impede the magnitude of the muscle response to training in this population. In this study, it is possible that heightened stiffness of the PFMs and surrounding connective tissues may have limited the displacement of the muscle that could be visualized on ultrasound. Nonetheless, UI improved quickly and regardless of small PFM changes, suggesting that changes may also have been derived from the bladder training and education in fluid management and bladder emptying techniques, for example. It is also plausible that the statistical tests used, despite their indication for very small sample sizes, may have been underpowered to detect changes in these outcomes.

This single-case experimental study had many strengths. First, by prospectively measuring change through three distinct phases, this study provides high internal validity. By replicating the design with eight participants of different ages, body mass indices and levels of ease with technology, the results indicate that this approach is suitable for different types of survivors of EC. Moreover, caution was applied with the selection criteria for this study: eligible women were at least 12 months post-treatment, which made it improbable that their UI would have spontaneously recovered over the duration of the program [4, 30, 31]. Also, the assessor for the main outcome was blinded to the intervention phase, limiting risk of bias. The main limitation of this study is the difficulty in producing stable pad test results during the baseline phase. The variability of the results, even though a standardized approach was observed, could be explained by the possibility that leakage may not be as reproducible in women with symptoms of mixed UI, especially with a predominance of urge symptoms. Although evidence on this topic is scarce, it has been proposed before that leakage can be more variable in women with urgency and mixed UI than in women with stress UI [32]. Moreover, leakage in circumstances of urgency may be triggered in specific situations only, which may not be reproduced by the standardized tasks included in the 1-h pad test. Also, it is possible that EC survivors with UI may not respond similarly to women with comparable symptoms during this test because of differences in the etiology of their urinary symptoms. Lastly, even though *n* = 8 participants is an adequate sample size for a single-case experimental design, the design itself limits the generalization of the results to all women who experience UI after EC treatments.

In conclusion, our findings support an in-home rehabilitation approach, including the use of mobile technology and remote supervision by a physiotherapist, to treat UI in survivors of EC. This mode of delivery has the potential to address a gap in access to pelvic floor physiotherapy services for those living in rural and remote communities. Further work is needed to explore the benefits as well as to determine the optimal dosage and mode of delivery given the high prevalence of UI among EC survivors.

## Declarations

Chiaro Technology Limited provided a discount to the research team for the acquisition of the Elvie Trainer devices.
